# CMPD1 inhibited human gastric cancer cell proliferation by inducing apoptosis and G2/M cell cycle arrest

**DOI:** 10.1186/s40659-018-0159-6

**Published:** 2018-04-16

**Authors:** Yu Li, Depeng Zhang, Kaikai Yu, Yudong Hu, Qiong Wu, Feng Qian, Zishu Wang

**Affiliations:** 1 Department of Medical Oncology, The First Affiliated Hospital of Bengbu Medical College, 287 Changhuai Road, Bengbu, 233004 Anhui People’s Republic of China; 2 Center for Cancer Precision Medicine, Bengbu Medical College, Bengbu, 233003 Anhui People’s Republic of China; 3 Engineering Research Center of Cell, & Therapeutic Antibody, Ministry of Education, School of Pharmacy, Shanghai Jiao Tong University, Shanghai, People’s Republic of China

**Keywords:** CMPD1, Proliferation, Apoptosis, Cell cycle arrest, MKN-45

## Abstract

**Background:**

Gastric cancer occupies the fourth highest morbidity rate of cancers worldwide. Clinical therapies of gastric cancer remain limited because of uncertainty of mechanisms and shortness of effective medicine. Thus, new drug candidates for gastric cancer treatment is urgently needed.

**Results:**

In this study, CMPD1 as a wildly used MK2 phosphorylation inhibitor was employed to find its impact on gastric cancer cell proliferation, apoptosis and cell cycle using colony formation assay and flow cytometry analysis. Along with its anti-proliferation effect on gastric cancer cell line MKN-45 and SGC7901, CMPD1 also induced massive apoptosis and significant G2/M phase arrest in a time-dependent and dose-dependent manner in MKN-45 cells respectively. Furthermore, Western blot confirmed that the expression of anti-apoptotic proteins Bcl-2 was decreased while BAX, cytochrome c release and cleaved PARP were increased. In addition, oncogene c-Myc was downregulated in response to CMPD1 treatment.

**Conclusions:**

Our results demonstrated that CMPD1 has anti-tumor effect on human gastric cancer cell line MKN-45 possibly via downregulating oncogene c-Myc expression and CMPD1 could be applied as a potential candidate for treating gastric malignancy. To the best of our knowledge, it is the first report of anti-tumor effect of CMPD-1 on human gastric cancer cells.

## Background

With poor prognosis and high morbidity, gastric cancer remains the second leading cause of cancer-related death and ranks the fourth in common types of malignancies worldwide [1, 2]. Particularly in China, gastric cancer occupies the second highest mortality and incidence rate [3]. Despite the decreased mortality rates and the advanced approaches to cure this illness, treatment of patients with gastric cancer is still limited with disappointing outcomes [4]. Among those, surgical management combined with chemotherapy and radiation therapies is the most effective solution clinically [5, 6]. Nevertheless, being major problems in early gastric cancer, radical resection, postoperative recurrence and metastasis hinder the curative effect that current treatment could achieve [4]. Thus, potentially effective strategies and new curative candidates are strongly demanded in the upcoming years with an aging population worldwide [7].

Like other cancers, the occurrence of gastric cancer is the outcome of sequential genetic and epigenetic changes with resistance to DNA damage as one of the hallmarks [8–10]. Among all the complexed factors that may trigger gastric carcinoma development, DNA damage response (DDR) regulating cell cycle at G1/S, intra-S phase and G2/M phase play a pivotal role for DNA repairing and cell survival. Mechanistic studies revealed two canonical DDR signaling pathways which compromise kinases ATR and ATM with their downstream effectors Chk1 and Chk2 respectively [11]. Deficiency of Chk1 in DT40 cells has highlighted the necessity of Chk1 in DNA damage-induced G2 arrest while Chk2 is required in the intra-S phase checkpoint during DDR [12].

p38 mitogen-activated protein kinase (MAPK) with its substrate MAPK-activated protein kinase 2 (MK2) was involved in a third DDR effector pathway, which was confirmed lately [13]. p38 MAPK/MK2 are found in most cells affecting cell proliferation, differentiation, apoptosis, inflammation and oxidative stress by coordinating the expression of its related effector molecules such as c-Myc, Bcl-2 family proteins and p53 [14, 15]. Numerous studies have found that MK2 mediates RNA stabilization, phosphorylation of Cdc25 and prevents Myc-dependent DNA replication [16, 17] as well as resulting in G2/M arrest and DNA repair [18]. Although massive apoptosis has been observed under the circumstance that MK2 is inhibited in p53-deficient cancer [19] or combined with Chk1 inhibition in KRAS-mutant cancer [20], whether the inhibition of MK2 alone could exert such anti-tumor effect on human gastric cancer has not been illustrated.

CMPD1, a selective p38 MAPK/MK2 inhibitor, was developed as a non-ATP competitive inhibitor with apparent Ki of 330 nM [21]. To our best knowledge, few researches reported applying CMPD1 against tumor cells [22] while the application of CMPD1 in treatment of gastric cancer still remains unveiled. In this study, we investigated whether using CMPD1 as a MK2a inhibitor alone has cytotoxic effect in human gastric cancer cells. We found that CMPD1 could largely inhibit cell growth and remarkably induce cell apoptosis with expression alternations in apoptotic-related proteins such as Bcl-2, Bax and cytochrome c release. Furthermore, we revealed that CMPD1 caused a dose-dependent cell cycle arrest which mainly occurred in the G2/M phase possibly due to oncogene c-Myc downregulation. Thus, our results indicate that CMPD1 has anti-tumor effect on human gastric cancer cell line MKN-45 possibly via downregulating oncogene c-Myc expression. These observations revealed and evidenced the potential value CMPD1 contains in curing gastric malignancy.

## Methods

### Cell culture and reagents

MKN-45 gastric cancer cells were obtained from American Type Culture Collection (ATCC; Rockville, MD, USA). SGC7901 gastric cancer cells were provided by the cell bank of the Chinese Academy of Sciences (Shanghai, China). Fetal bovine serum (FBS) was purchased from Hangzhou Sijiqing Biological Engineering Materials Co., Ltd. (Hangzhou, China). RPMI-1640 and penicillin/streptomycin were purchased from Gibco (Grand Island, NY, USA). Cells were routinely cultivated in RPMI-1640 supplemented with 10% heat-inactivated FBS and 100U/ml penicillin/streptomycin (100 mg/ml) in humidified incubator at 37 °C, 5% CO_2_. CMPD1 was purchased from Tocris Bioscience (Bristol, UK). Dimethyl sulfoxide (DMSO) was purchased from Sigma-Aldrich (St. Louis, MO, USA). GIemsa dye was obtained from Nan Jing Jian Cheng Technology Company (Nanjing, China). Annexin V/PI apoptosis kit was provided by BestBio (Shanghai, China). c-Myc, cleaved PARP, GAPDH, BAX and Bcl-2 first antibodies were purchased from Santa Cruz Biotechnology (Dallas, TX, USA). Anti-cytochrome c antibody was purchased from Cell Signaling Technologies (Danvers, MA, USA).

### Colony formation assay

SGC7901 and MKN-45 gastric cancer cells in exponential growth period were grown in six-well plates (300 cells per well). The cells treated with CMPD1 at various concentrations (30,100,300 nM) were incubated in 2 ml of RPMI 1640 supplemented with 10% FBS and were incubated at 37 °C and 5% CO_2_ for 7–10 days until they reached optimal clones of 200 cells in each clone. DMSO at a final concentration of 0.1% was added as a control group. In the end of the incubation, cells were fixed with methanol followed by staining with GIemsa dye. Finally, the clones contained more than 200 cells were counted and averaged. IMAGE J software (Software Inquiry, Quebec, Canada) was used to calculate grey value and the colony formation rate in each well.

### Cell apoptosis assay

The cells (5 × 10^5^) supplemented with 10% FBS were seeded in six-well plates and treated with CMPD1 at various concentrations (0.3, 1, 3, 10 μM) for 24 and 48 h. Cells were harvested and rinsed with cold PBS twice, then incubated with an Annexin V-FITC and PI apoptosis detection kit. Samples were kept on ice and analyzed on flow cytometry (LSRFortessa TM X-20; BD Biosciences, San Jose, NJ, USA). The data were analyzed using FLOWJO7.6 software (Treestar, Ashland OR, USA).

### Cell cycle assay

Cells (5 × 10^5^) supplemented with 10% FBS were cultured in six-well plates and incubated with vehicle, various concentrations of CMPD1 (0.1, 0.3, 1.0 μM) for 24 h. When cells were harvested, the medium was discarded. Cells were digested by 0.25% trypsin (Gibco, NY, USA) and were dispersed thoroughly. Then, cells were collected and rinsed with cold PBS for twice before being fixed in cold 70% ethanol at 4 °C overnight. After removal of ethanol by centrifugation, 10 μg/ml RNAse was added to the fixed cells and incubated at 37 °C for 30 min. In the end, cells were stained with 50 μg/ml PI for 5 min in the dark. Samples were analyzed by flow cytometry (LSRFortessa TM X-20; BD Biosciences, San Jose, NJ, USA).

### Preparation of cytosolic fractions

To determine the cytochrome c release from mitochondria to cytosol, cytosolic fractions was prepared using a commercial Cell Mitochondria Isolation Kit (Beyotime, Beijing, China). Briefly, cells were harvested and incubated in 150 μl pre-cold mitochondrial lysis buffer on ice for 10 min. Then cells were homogenized for 25 times using a glass homogenizer with a tight pestle on ice before being subjected to centrifugation at 600*g* at 4 °C for 10 min to remove nuclei and unbroken cells. The supernatant was carefully collected and subjected to centrifugation at 11,000*g* for 10 min. The supernatant after centrifugation was collected again and centrifuged at 12,000*g* for 10 min, the final supernatant containing cytosolic fractions were dissolved in loading buffer and proteins were analyzed by Western blot.

### Western blot

Cells (1 × 10^6^) grown in six-well plates were incubated at 37 °C for 24 h with CMPD1 treatment at various concentrations. Then cells were digested with 0.25% trypsin and washed with cold PBS twice. Protein was extracted using RIPA buffer with 1 mM PMSF. Protein lysates were heated in 99 °C for 10 min before being mixed evenly and centrifuged slightly. The proteins were separated by SDS-PAGE electrophoresis and transferred to nitrocellulose membranes followed by blocking for 2 h with 5% nonfat milk dissolved in water. The membranes were incubated with primary antibodies (cleaved PARP, Bax, Bcl-2, c-Myc, GAPDH, cytochrome c and β-actin) overnight at 4 °C. Then the membranes were incubated with fluorescent antibodies at room temperature for 2 h. After being washed, the bound antibodies were detected by the ECL Western blot detection system (Thermo Scientific, Rockford, USA). Quantification of Western blot was performed using ImageJ software.

### Data analysis and statistics

Data were represented as mean ± SEM, analysis was performed using statistical methods including Student’s T test. Statistical analyses were performed using GraphPad prism 5 (GraphPad, San Diego, CA, USA). Statistically significant P-values were defined as *P < 0.05 and **P < 0.01, ***P < 0.005.

## Results

### The impact of CMPD1 on cell proliferation

**Fig. 1:**
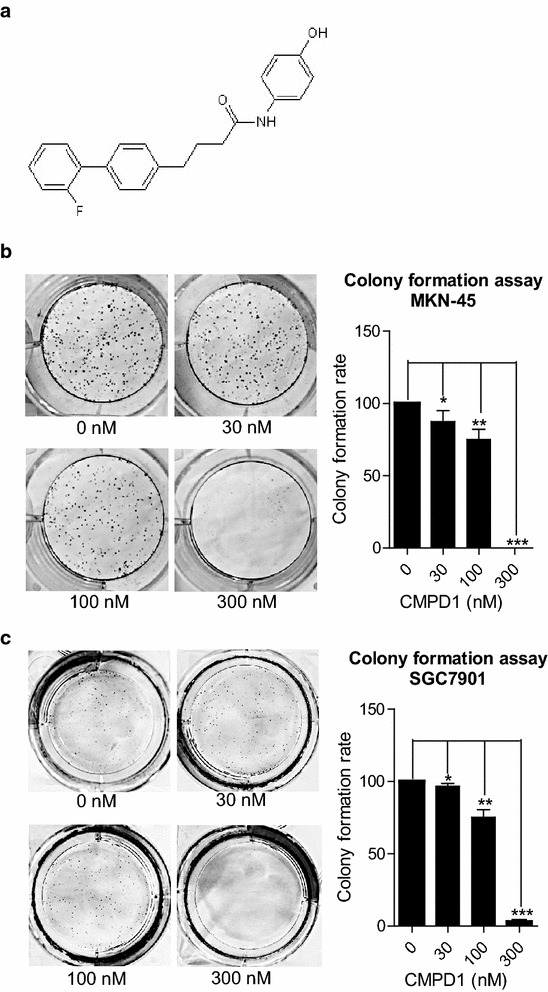
The chemical structure of CMPD1 and its inhibitory effect on gastric tumor MKN-45 and SGC7901 cell proliferation. **a** Chemical structure of CMPD1. Representative images of colonies and quantification of the colony formation rate in **b** MKN-45 and **c** SGC7901 cells from a six-well plate using colony formation assay. Cells were treated with 0, 30, 100 and 300 nM of CMPD1 respectively. *P < 0.05, **P < 0.01 and ***P < 0.001 vs. control

The chemical structure of CMPD1 was shown in Fig. 1a. Colony formation assay was used to determine the anti-proliferative effect of CMPD1 in human gastric cancer MKN-45 and SGC7901 cells at various doses. As shown in the Fig. 1b, c, the number of MKN-45 and SGC7901 cell colonies underwent a significant decrease when treated with CMPD1 for 7–10 days. Quantification of the colony formation rate revealed that CMPD1 suppressed proliferation capacity of MKN-45 and SGC7901 cell in a dose-dependent manner.

### CMPD1 induces apoptosis in MKN-45 cells

**Fig. 2:**
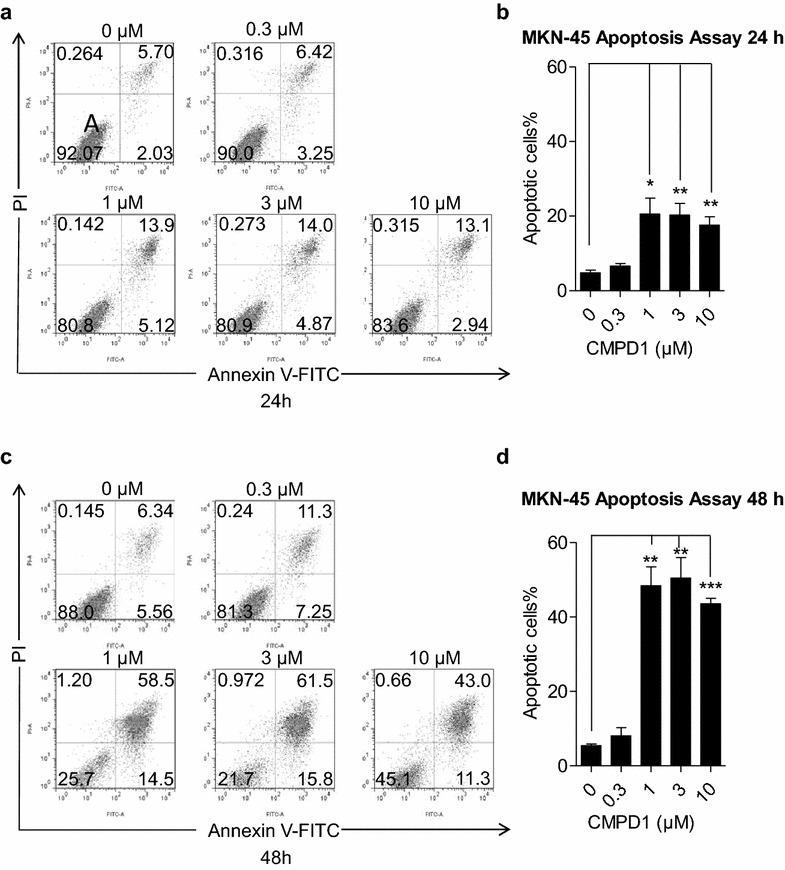
CMPD1 promoted apoptosis in MKN-45 cells. The upper-left, upper-right, lower-left, lower-right quadrants indicated necrotic, late apoptotic, viable and early apoptotic cell population, respectively. MKN-45 cells were treated with 0, 0.3, 1, 3 and 10 μM of CMPD1 respectively for **a** 24 h and **c** 48 h, and were then subjected to Annexin V-FITC/PI staining, followed by flow cytometer analysis. Quantification of the percentage of apoptotic cells treated with CMPD1 at various doses after treatment for **b** 24 h and **d** 48 h. *P < 0.05, **P < 0.01 and ***P < 0.001 vs. control

We further investigated whether CMPD1 inhibited cell proliferation by inducing apoptosis in MKN-45 cells. The cells treated with or without CMPD1 were subjected to Annexin V-FITC/PI double staining, followed by flow cytometry analysis. As shown in Fig. 2a, CMPD1-treated groups with 24 h displayed a late apoptosis in 6.42, 13.9, 14 and 13.1% of the cells with 0.3, 1, 3, 10 μM of CMPD1, respectively. Furthermore, after treatment with CMPD1 for 48 h, apoptosis rate of MKN-45 cells increased to 11.3, 58.5, 61.5 and 43% at different doses from 0.3 to 10 μM, reflecting a time-dependent effect of CMPD1-caused cell apoptosis. Statistical analysis showed that CMPD1 significantly induced MKN-45 cell apoptosis at the concentration of 1, 3 and 10 μM for 24 and 48 h respectively (Fig. 2b, d).

### CMPD1 downregulates BCL-2 and c-Myc while upregulating BAX, cytochrome c release and cleaved PARP

**Fig. 3:**
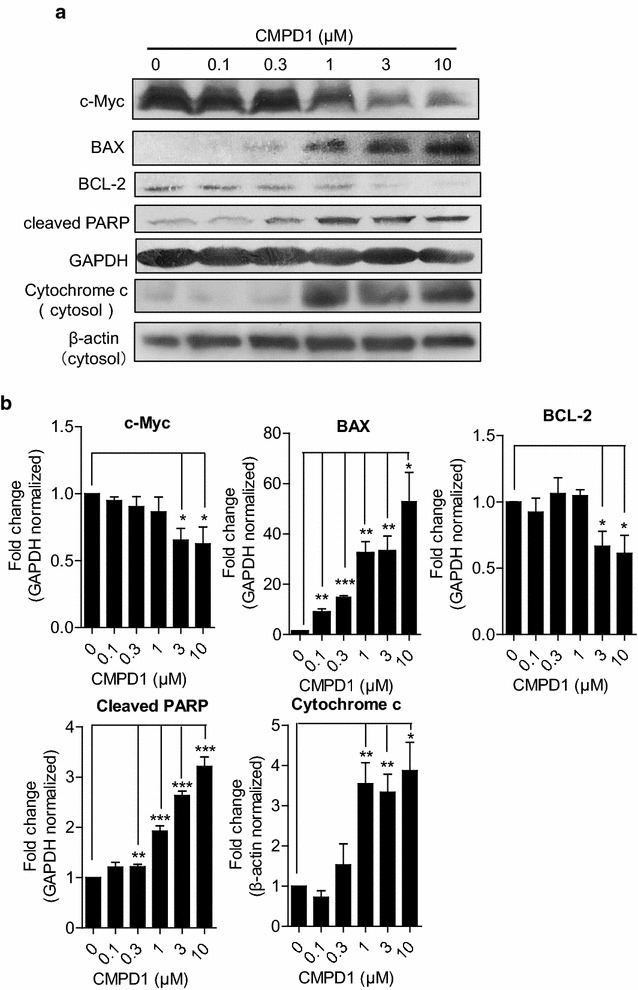
CMPD1 induced gastric cancer cell apoptosis by suppressing the expression of c-Myc and Bcl-2. **a** After treatment of MKN-45 cell line with indicated concentration of CMPD1 for 24 h, the expression of cleaved PARP, c-Myc, Bax, Bcl-2 and cytochrome c release were analyzed by western blot, GAPDH and β-actin were used as internal controls. **b** The ratio of cleaved PARP, c-Myc, Bax and Bcl-2 to GAPDH and cytosolic cytochrome c to β-actin was performed in densitometry analyzed by IMAGE J software. Data are expressed using Student’s test, *P < 0.05, **P < 0.01 and ***P < 0.001

Western blot analysis of apoptosis-related proteins was applied for supporting the fact that CMPD1 induces apoptosis in MKN-45 cells. Figure 3a shows a representative image of the expression of oncogene c-Myc, anti-apoptotic protein BCL-2 and pro-apoptotic signals BAX, cleaved PARP and cytochrome c release in MKN-45 cells. Compared with the internal control, expression of BAX, cleaved PARP and the release of cytochrome c were significantly upregulated accompanied by BCL-2 and c-Myc downregulation under the impact of CMPD1 in a dose-dependent way, which indicated that CMPD1 induced cell death through BAX and BCL-2 mediated apoptosis. The quantification ratio of c-Myc, BAX, BCL-2 and cleaved PARP to GAPDH and cytosolic cytochrome c to β-actin are shown respectively in Fig. 3b.

### CMPD1 treatment induces G2/M cell cycle arrest in MKN-45 cells

**Fig. 4:**
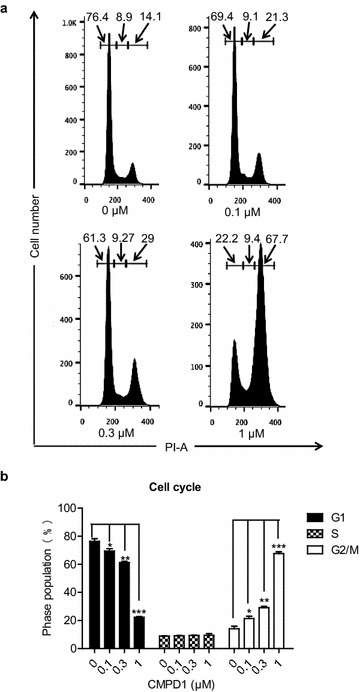
CMPD1 induced G2/M cell cycle arrest in MKN-45 cells. **a** Human gastric cancer cell line MKN-45 cells were treated with 0, 0.1, 0.3, and 1.0 μM of CMPD1 respectively for 24 h, and cell cycle distribution of MKN-45 was analyzed by flow cytometry. **b** The proportion of cells in G1, S, G2/M phase was exhibited from three independent experiments. CMPD1 significantly arrested cell cycle at G2/M phase in a dose-dependent manner. *P < 0.05, **P < 0.01 and ***P < 0.001 vs. control

We next explored the impact of CMPD1 on cell cycle arrest in MKN-45 cells. The distribution of cell cycle phase was demonstrated by using flow cytometry analysis. As shown in Fig. 4a, compared to the control group which showed only 14.1% of cells in G2/M phase, the proportion of G2/M cells in 0.1, 0.3 and 1 μM-CMPD1 treated cells increased to 21.3, 29 and 67.7% respectively. The increased tendency of cell proportion in G2/M phase was accompanied by a decrease of cell percentage in the G1 phase, indicating that CMPD1 inhibits cell proliferation by inducing G2/M cell cycle arrest. Figure 4b shows a graphical representation of the CMPD1-induced change of cell proportion in G1 phase, S phase and G2/M phase.

## Discussion

Our findings demonstrated that CMPD1 exerted great anti-proliferation on human gastric cancer MKN-45 and SGC7901 cells. Meanwhile, its apoptotic activity in MKN-45 cells was also disclosed with elevated pro-apoptotic protein BAX and cytochrome c release. Accordingly, cell cycle G2/M arrest with downregulation of c-Myc expression is responsible for the fate of apoptosis.

Colony formation assay is a cell survival assay which based on the ability of single cells to proliferate indefinitely to grow into colonies in vitro. This assay determines cell reproductive death under radiation or cytotoxic agents-treatment and is widely used for evaluating cytotoxicity induced by chemotherapeutic candidates [23]. Although CMPD1 inhibited cell proliferation at certain concentrations (1–5 μM) in studied glioblastoma cells [22], it totally abrogated proliferation capacity of gastric cell lines MKN-45 and SGC7901 at a very low concentration (300 nM), indicating a better sensitivity of gastric cancer cells to CMPD1 treatment. Furthermore, apoptosis at a low concentration (1 μM) was also observed at 24 and 48 h accompanied by drastic G2/M checkpoint arrest. Hence, we hypothesize that sensitivity to cell death caused by CMPD1 differed from the subtype of cancer cell. Further study is in need for identifying the specific cancer cell type with optimal drug therapy.

Of all the pathways directly influence cell death, TNF-related apoptosis-inducing ligand (TRAIL) apoptotic pathway and mitochondrial apoptotic pathway stand for the canonical extrinsic and intrinsic death pathway, respectively [24]. Bcl-2 family proteins including anti-apoptotic Bcl-2 and pro-apoptotic BAX are important regulators during mitochondria-mediated apoptosis [25, 26]. The latter one causes apoptotic cytochrome c release from the mitochondria to the cytoplasm with further activating caspase 9/caspase 3 cascade [27, 28]. While cleaved PARP is assumed as death substrate during caspase cascade during apoptosis [29, 30]. Our results showed decreased Bcl-2 and increased BAX and cytochrome c release as well as cleaved PARP due to the induction of CMPD1 at various concentrations, which is corresponding to the current canonical mitochondrial apoptotic pathway. Thus, we proposed that CMPD1 might induce cell apoptosis through intrinsic death pathway which often occurs in DNA damage response [31, 32].

Inhibitors targeting checkpoints or agents like taxanes targeting microtubules enjoy most success for anticancer treatment [33, 34]. However, overall cytotoxicity hinders their development and clinical use [35]. MK2 is recently identified as a novel controller involved in cell cycle progression [36]. MK2 depleted cell are more vulnerable to irradiation followed by checkpoint dysfunction at G1/S and G2/M [37]. Clinically, an inhibitor against MK2 was announced to have synergistic anticancer effect combined with cisplatin [38]. While our data further demonstrated MK2a inhibition alone would cause dramatic G2/M arrest and apoptosis, remarkable suppression of c-Myc expression could not be ignored. As previous research has revealed that c-Myc protein expression occurred in G1 and G2 phase with rapid increase when cell cycle was re-initiated [39]. And recent evidence has shown that c-Myc may attenuate G2/M arrest induced by DNA damage [40–42]. Based on these findings, we proposed an insight into the relationship between CMPD1-induced cell cycle G2/M arrest and c-Myc downregulation.

It is commonly assumed that with a combination use of chemotherapeutic drugs, greater cytotoxic effect could be anticipated, as a recent study revealed that combination of paclitaxel and cisplatin induced more cell death in MKN-45 cell line [43]. Thereby, the role of combination of CMPD1 with chemotherapeutic agents in viable cancer cells deserves to be further investigated. Especially given the fact that CMPD1 as non-ATP competitive inhibitor with a high selectivity of MK2a may lead to less side effects possibly due to fewer downstream signals MK2 contains [21, 44, 45].

## Conclusions

This study highlights the anti-tumor effect CMPD1 displays in human gastric cancer cell and is the first report to indicate that CMPD1 may inhibit cell proliferation and induce G2/M arrest through downregulating oncogene c-Myc expression. Taken together with the other finding that reports cytotoxic activity of CMPD1 on glioblastoma cells [22], this study further confirmed the potential efficacy and feasibility CMPD1 possesses to be applied as a clinical candidate for gastric cancer treatment. Finally, detailed mechanism as well as adverse effects of CMPD1 should be explored to disclose its pharmacology studies before clinical trials.

## Abbreviations

DDR: DNA damage response
MAPK: mitogen-activated protein kinase
MK2: MAPK-activated protein kinase 2
FBS: fetal bovine serum
DMSO: dimethyl sulfoxide
TRAIL: TNF-related apoptosis-inducing ligand
